# 548. Convalescent Plasma in Hospitalized Pediatric and Obstetric patients with COVID-19

**DOI:** 10.1093/ofid/ofab466.747

**Published:** 2021-12-04

**Authors:** Saki Ikeda, Eduardo Benzi, Lisa Hensch, Sridevi Devaraj, Shiu-Ki Rocky Hui, Manisha Gandhi, Karin Fox, Jun Teruya, Flor M Munoz

**Affiliations:** 1 Baylor College of Medicine/Texas Children's Hospital, Houston, Texas; 2 Baylor College of Medicine, Houston/Texas Children's Pavilion for Women, Houston, Texas; 3 Baylor College of Medicine/Texas Children's Pavilion for Women, Houston, Texas; 4 Baylor College of Medicine, Houston, Texas

## Abstract

**Background:**

Published data on COVID-19 convalescent plasma (CCP) use in children and obstetric patients is limited. We describe a single-center experience of hospitalized patients who received CCP for acute COVID-19.

**Methods:**

We performed a retrospective review of children 0-18-years-old and pregnant patients hospitalized with laboratory-confirmed acute COVID-19 who received CCP from March 1st, 2020 to March 1st, 2021. Clinical and laboratory data were collected to assess the safety of CCP administration. Antibodies to SARS-CoV-2 were measured before and at various timepoints post CCP transfusion. Correlation between SARS-CoV-2 immunoglobulin administered versus the SARS-CoV-2 anti-Spike immunoglobulin response in patient serum was assessed.

**Results:**

Twenty-two children and 10 obstetric patients were eligible. 12 pediatric and 8 obstetric patients had moderate disease and 10 pediatric and 2 obstetric patients had severe disease. 5 pediatric patients died. 18/37 (48.6%) CCP units that were measured met FDA criteria for a high IgG titer. There were no complications with transfusion based on CDC, NHSN Biovigilance Component: Hemovigilance Module Surveillance Protocol. Two pediatric patients had fevers a few hours after CCP with low suspicion for a transfusion reaction. Median SARS-CoV-2 anti-spike antibody levels of pediatric patients post-transfusion for 0-7 days was 80.6AU/mL (range: 2-1070), 8-21 days was 180AU/mL (range: 12-661) and >21 days was 210AU/mL (range: 4.1-1220). For obstetric patients, post-transfusion antibody levels were only obtained 0-7 days post-transfusion with median 45AU/mL (range: 9.5-100). High-titer CCP showed a positive correlation with rise in patient immunoglobulin levels only in the obstetric patients but not in pediatric patients.

**Conclusion:**

CCP was administered safely to our moderately to severely ill pediatric and obstetric patients. Among pediatric patients, the median serum antibody level increased over time after transfusion and suggested that CCP did not interfere with the endogenous antibody production. Antibody dose of high-titer CCP correlated with post-transfusion response in only obstetric patients. Randomized trials in pediatric and obstetric patients are needed to further understand how to dose CCP and evaluate efficacy.

**Disclosures:**

**Jun Teruya, MD, PhD**, **Apelo Consulting Pvt. Ltd** (Consultant)**Hemosonics** (Other Financial or Material Support, Honorarium) **Flor M. Munoz, MD**, **Biocryst** (Scientific Research Study Investigator)**Gilead** (Scientific Research Study Investigator)**Meissa** (Other Financial or Material Support, DSMB)**Moderna** (Scientific Research Study Investigator, Other Financial or Material Support, DSMB)**Pfizer** (Scientific Research Study Investigator, Other Financial or Material Support, DSMB)**Virometix** (Other Financial or Material Support, DSMB)

